# Dichlorido(pyridine-κ*N*)[2-(pyridinium-1-yl)acetato-κ*O*]zinc(II)

**DOI:** 10.1107/S1600536812011749

**Published:** 2012-03-28

**Authors:** Zhang Qun, Chen Jin-Xiang

**Affiliations:** a The Third Affiliated Hospital of Southern Medical University, Guangzhou 510515, Guangdong, People’s Republic of China; b School of Pharmaceutical Science, Southern Medical University, Guangzhou 510515, Guangdong, People’s Republic of China

## Abstract

In the title complex, [ZnCl_2_(C_5_H_5_N)(C_7_H_7_NO_2_)], the Zn^II^ atom adopts a distorted tetra­hedral coordination geometry [the smallest angle being 105.22 (15)° and the widest angle being 115.60 (16)°] that is formed from one monodentate carboxyl­ate ligand, one pyridine ligand and two Cl atoms.

## Related literature

For background to metalloenzymes, see: Holm & Solomon (2004 ▶), Karambelkar *et al.* (2002 ▶).
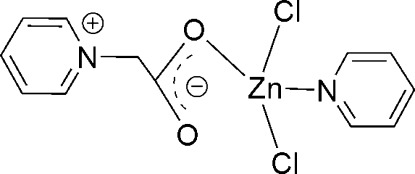


## Experimental

### 

#### Crystal data

[ZnCl_2_(C_5_H_5_N)(C_7_H_7_NO_2_)]
*M* *_r_* = 352.51
Monoclinic, 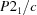

*a* = 9.979 (2) Å
*b* = 13.462 (3) Å
*c* = 13.900 (5) Å
β = 128.781 (19)°
*V* = 1455.6 (7) Å^3^
*Z* = 4
Mo *K*α radiation
μ = 2.05 mm^−1^
*T* = 291 K
0.48 × 0.36 × 0.32 mm

#### Data collection

Rigaku SCXmini diffractometer
Absorption correction: multi-scan (*REQAB*; Jacobson, 1998 ▶) *T*_min_ = 0.439, *T*_max_ = 0.560
14915 measured reflections
3330 independent reflections
2758 reflections with *I* > 2σ(*I*)
*R*_int_ = 0.041

#### Refinement

*R*[*F* ^2^ > 2σ(*F* ^2^)] = 0.073
*wR*(*F* ^2^) = 0.233
*S* = 1.05
3330 reflections
172 parameters
H-atom parameters constrained
Δρ_max_ = 1.96 e Å^−3^
Δρ_min_ = −1.22 e Å^−3^

### 

Data collection: *CrystalClear* (Rigaku, 2005 ▶); cell refinement: *CrystalClear*; data reduction: *CrystalStructure* (Rigaku/MSC, 2004 ▶); program(s) used to solve structure: *SHELXS97* (Sheldrick, 2008 ▶); program(s) used to refine structure: *SHELXL97* (Sheldrick, 2008 ▶); molecular graphics: *SHELXTL/PC* (Sheldrick, 2008 ▶); software used to prepare material for publication: *SHELXTL/PC* and *PLATON* (Spek, 2009 ▶).

## Supplementary Material

Crystal structure: contains datablock(s) I, global. DOI: 10.1107/S1600536812011749/ff2059sup1.cif

Structure factors: contains datablock(s) I. DOI: 10.1107/S1600536812011749/ff2059Isup2.hkl

Additional supplementary materials:  crystallographic information; 3D view; checkCIF report

## Figures and Tables

**Table 1:** Selected bond lengths (Å)

Zn1—O1	1.986 (5)
Zn1—N2	2.054 (5)
Zn1—Cl1	2.2884 (18)
Zn1—Cl2	2.2908 (15)

**Fig. 1.**
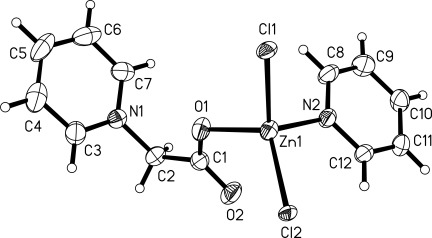
Ellipsoid plot of complex (I) at the 30% probability level. Hydrogen atoms are drawn as spheres of arbitrary radii.

## supplementary crystallographic information

### Comment

The study of synthetic active site analogues has made vast contribution to the understanding of the structure function relationship of many metalloenzymes (Holm *et al.*, 2004). Since the coordination environment of many metalloenzyme active sites is made up of different donor groups, the interest of synthetic chemists has shifted toward the design of mixed ligands (Karambelkar *et al.*, 2002). The title complex (I), has been prepared with the aim to mimic the structures and functions of the active sites of zinc metalloenzymes by using carboxylate ligand and pyridine ligand.

Complex (I) crystallizes in the monoclinic space group P21/c and the asymmetric unit contains one [C_12_H_12_Cl_2_N_2_O_2_Zn] molecule (Figure 1). In the complex (I), the Zn atom is coordinated one monodentate carboxylate ligand, one pyridine group and two Cl atoms, hence forming a distorted tetrahedral geometry.

### Experimental

The title complex was synthesized by reaction of *N*-(carboxymethyl)pyridinium bromide (1.09 g, 5 mmol) and ZnCl_2_ (0.68 g, 5 mmol) in pyridine (10 ml). The solution was stirred for 2 h to afford white precipitates. The precipitates were collected by filtration, re-dissolved in H_2_O (5 ml) then allowed to stand for several days to produce white crystals (I). Yield: 1.53 g (87%). The crystal used for the crystal structure determination was obtained directly from the above preparation. Analysis, found: C, 40.32; H, 3.31; N, 7.62%. calculated. for C_12_H_12_Cl_2_N_2_O_2_Zn: C, 40.76; H, 3.71; N, 7.92%.

### Refinement

Carbon-bond H atoms were positioned geometrically (C—H = 0.93 Å for phenyl group), and were included in the refinement in the riding model approximation, with *U*_iso_(H) = 1.2*U*_eq_(C).

### Crystal data

**Table:**

[ZnCl_2_(C_5_H_5_N)(C_7_H_7_NO_2_)]	*F*(000) = 712
*M**_r_* = 352.51	*D*_x_ = 1.609 Mg m^−^^3^
Monoclinic, *P*2_1_/*c*	Mo *K*α radiation, λ = 0.71073 Å
Hall symbol: -P 2ybc	Cell parameters from 15259 reflections
*a* = 9.979 (2) Å	θ = 3.0–27.5°
*b* = 13.462 (3) Å	µ = 2.05 mm^−^^1^
*c* = 13.900 (5) Å	*T* = 291 K
β = 128.781 (19)°	Prism, colourless
*V* = 1455.6 (7) Å^3^	0.48 × 0.36 × 0.32 mm
*Z* = 4	

### Data collection

**Table:**

Rigaku SCXmini diffractometer	3330 independent reflections
Radiation source: fine-focus sealed tube	2758 reflections with *I* > 2σ(*I*)
Graphite monochromator	*R*_int_ = 0.041
ω scans	θ_max_ = 27.5°, θ_min_ = 3.0°
Absorption correction: multi-scan (*REQAB*; Jacobson, 1998)	*h* = −12→12
*T*_min_ = 0.439, *T*_max_ = 0.560	*k* = −17→17
14915 measured reflections	*l* = −18→18

### Refinement

**Table:**

Refinement on *F*^2^	Primary atom site location: structure-invariant direct methods
Least-squares matrix: full	Secondary atom site location: difference Fourier map
*R*[*F*^2^ > 2σ(*F*^2^)] = 0.073	Hydrogen site location: inferred from neighbouring sites
*wR*(*F*^2^) = 0.233	H-atom parameters constrained
*S* = 1.05	*w* = 1/[σ^2^(*F*_o_^2^) + (0.1207*P*)^2^ + 8.0068*P*] where *P* = (*F*_o_^2^ + 2*F*_c_^2^)/3
3330 reflections	(Δ/σ)_max_ < 0.001
172 parameters	Δρ_max_ = 1.96 e Å^−^^3^
0 restraints	Δρ_min_ = −1.22 e Å^−^^3^

### Special details

**Table:**

Geometry. All e.s.d.'s (except the e.s.d. in the dihedral angle between two l.s. planes) are estimated using the full covariance matrix. The cell e.s.d.'s are taken into account individually in the estimation of e.s.d.'s in distances, angles and torsion angles; correlations between e.s.d.'s in cell parameters are only used when they are defined by crystal symmetry. An approximate (isotropic) treatment of cell e.s.d.'s is used for estimating e.s.d.'s involving l.s. planes.
Refinement. Refinement of *F*^2^ against ALL reflections. The weighted *R*-factor *wR* and goodness of fit *S* are based on *F*^2^, conventional *R*-factors *R* are based on *F*, with *F* set to zero for negative *F*^2^. The threshold expression of *F*^2^ > σ(*F*^2^) is used only for calculating *R*-factors(gt) *etc*. and is not relevant to the choice of reflections for refinement. *R*-factors based on *F*^2^ are statistically about twice as large as those based on *F*, and *R*- factors based on ALL data will be even larger.

### Fractional atomic coordinates and isotropic or equivalent isotropic displacement parameters (Å^2^)

**Table:**

	*x*	*y*	*z*	*U*_iso_*/*U*_eq_	
Zn1	0.42997 (9)	0.41457 (6)	0.23620 (7)	0.0417 (3)	
Cl1	0.59745 (17)	0.39808 (11)	0.44591 (12)	0.0375 (4)	
Cl2	0.27820 (17)	0.56039 (10)	0.16703 (15)	0.0397 (4)	
O1	0.5887 (6)	0.3946 (4)	0.1974 (4)	0.0487 (11)	
O2	0.3636 (6)	0.3927 (4)	−0.0052 (5)	0.0609 (14)	
N1	0.8205 (7)	0.3545 (4)	0.1608 (5)	0.0430 (12)	
N2	0.2623 (6)	0.2967 (4)	0.1606 (5)	0.0418 (12)	
C1	0.5189 (8)	0.3845 (5)	0.0830 (6)	0.0420 (14)	
C2	0.6349 (8)	0.3582 (5)	0.0525 (6)	0.0465 (15)	
H2A	0.6007	0.2938	0.0122	0.056*	
H2B	0.6185	0.4065	−0.0058	0.056*	
C3	0.9172 (11)	0.4374 (6)	0.1957 (8)	0.0593 (19)	
H3A	0.8674	0.4955	0.1502	0.071*	
C4	1.0839 (12)	0.4372 (9)	0.2949 (10)	0.079 (3)	
H4A	1.1477	0.4954	0.3197	0.095*	
C5	1.1604 (11)	0.3512 (10)	0.3600 (8)	0.083 (3)	
H5A	1.2768	0.3496	0.4275	0.100*	
C6	1.0591 (13)	0.2657 (9)	0.3225 (8)	0.077 (3)	
H6A	1.1076	0.2061	0.3648	0.092*	
C7	0.8907 (11)	0.2704 (6)	0.2246 (7)	0.0570 (18)	
H7A	0.8224	0.2142	0.2010	0.068*	
C8	0.3213 (9)	0.2025 (6)	0.2002 (7)	0.0558 (18)	
H8	0.4388	0.1923	0.2601	0.067*	
C9	0.2123 (11)	0.1218 (6)	0.1544 (9)	0.064 (2)	
H9A	0.2561	0.0581	0.1825	0.077*	
C10	0.0402 (10)	0.1364 (6)	0.0676 (8)	0.0573 (18)	
H10A	−0.0353	0.0830	0.0373	0.069*	
C11	−0.0216 (9)	0.2317 (5)	0.0247 (7)	0.0493 (16)	
H11A	−0.1386	0.2431	−0.0361	0.059*	
C12	0.0944 (8)	0.3094 (5)	0.0742 (6)	0.0426 (14)	
H12A	0.0528	0.3734	0.0456	0.051*	

### Atomic displacement parameters (Å^2^)

**Table:**

	*U*^11^	*U*^22^	*U*^33^	*U*^12^	*U*^13^	*U*^23^
Zn1	0.0310 (4)	0.0442 (5)	0.0454 (5)	0.0012 (3)	0.0217 (4)	0.0033 (3)
Cl1	0.0289 (7)	0.0449 (8)	0.0263 (6)	0.0031 (5)	0.0112 (5)	0.0024 (5)
Cl2	0.0297 (7)	0.0281 (7)	0.0543 (9)	0.0099 (5)	0.0229 (6)	0.0156 (6)
O1	0.035 (2)	0.066 (3)	0.048 (3)	−0.003 (2)	0.027 (2)	−0.006 (2)
O2	0.036 (3)	0.069 (3)	0.055 (3)	−0.002 (2)	0.018 (2)	−0.003 (3)
N1	0.043 (3)	0.049 (3)	0.042 (3)	0.006 (2)	0.029 (2)	−0.001 (2)
N2	0.034 (3)	0.042 (3)	0.046 (3)	0.001 (2)	0.023 (2)	0.001 (2)
C1	0.038 (3)	0.035 (3)	0.048 (4)	−0.003 (2)	0.024 (3)	0.000 (3)
C2	0.042 (3)	0.047 (4)	0.041 (3)	0.003 (3)	0.021 (3)	−0.003 (3)
C3	0.056 (4)	0.060 (5)	0.065 (5)	−0.009 (4)	0.039 (4)	−0.003 (4)
C4	0.055 (5)	0.099 (8)	0.083 (7)	−0.012 (5)	0.043 (5)	−0.010 (6)
C5	0.037 (4)	0.156 (11)	0.053 (5)	0.008 (6)	0.026 (4)	−0.006 (6)
C6	0.077 (6)	0.105 (8)	0.058 (5)	0.047 (6)	0.047 (5)	0.031 (5)
C7	0.066 (5)	0.059 (5)	0.055 (4)	0.015 (4)	0.043 (4)	0.010 (3)
C8	0.041 (3)	0.051 (4)	0.064 (4)	0.015 (3)	0.027 (3)	0.013 (3)
C9	0.068 (5)	0.039 (4)	0.084 (6)	0.009 (4)	0.047 (5)	0.010 (4)
C10	0.058 (4)	0.044 (4)	0.076 (5)	−0.008 (3)	0.045 (4)	−0.005 (4)
C11	0.041 (3)	0.053 (4)	0.056 (4)	−0.006 (3)	0.030 (3)	−0.004 (3)
C12	0.035 (3)	0.044 (3)	0.043 (3)	0.004 (3)	0.022 (3)	0.006 (3)

### Geometric parameters (Å, º)

**Table:**

Zn1—O1	1.986 (5)	C4—C5	1.370 (16)
Zn1—N2	2.054 (5)	C4—H4A	0.9300
Zn1—Cl1	2.2884 (18)	C5—C6	1.399 (16)
Zn1—Cl2	2.2908 (15)	C5—H5A	0.9300
O1—C1	1.283 (8)	C6—C7	1.347 (12)
O2—C1	1.240 (8)	C6—H6A	0.9300
N1—C3	1.352 (10)	C7—H7A	0.9300
N1—C7	1.334 (9)	C8—C9	1.379 (12)
N1—C2	1.485 (8)	C8—H8	0.9300
N2—C12	1.327 (8)	C9—C10	1.359 (12)
N2—C8	1.360 (9)	C9—H9A	0.9300
C1—C2	1.502 (9)	C10—C11	1.386 (11)
C2—H2A	0.9700	C10—H10A	0.9300
C2—H2B	0.9700	C11—C12	1.381 (9)
C3—C4	1.339 (12)	C11—H11A	0.9300
C3—H3A	0.9300	C12—H12A	0.9300
			
O1—Zn1—N2	106.8 (2)	C5—C4—H4A	120.0
O1—Zn1—Cl1	105.22 (15)	C3—C4—H4A	120.0
N2—Zn1—Cl1	106.73 (16)	C4—C5—C6	118.4 (8)
O1—Zn1—Cl2	115.60 (16)	C4—C5—H5A	120.8
N2—Zn1—Cl2	109.57 (15)	C6—C5—H5A	120.8
Cl1—Zn1—Cl2	112.40 (7)	C7—C6—C5	119.4 (9)
C1—O1—Zn1	116.4 (4)	C7—C6—H6A	120.3
C3—N1—C7	120.2 (7)	C5—C6—H6A	120.3
C3—N1—C2	119.6 (6)	N1—C7—C6	121.0 (9)
C7—N1—C2	120.3 (7)	N1—C7—H7A	119.5
C12—N2—C8	117.9 (6)	C6—C7—H7A	119.5
C12—N2—Zn1	121.7 (5)	N2—C8—C9	122.1 (7)
C8—N2—Zn1	120.4 (5)	N2—C8—H8	118.9
O2—C1—O1	126.0 (6)	C9—C8—H8	118.9
O2—C1—C2	116.7 (6)	C8—C9—C10	119.2 (7)
O1—C1—C2	117.3 (6)	C8—C9—H9A	120.4
N1—C2—C1	114.5 (5)	C10—C9—H9A	120.4
N1—C2—H2A	108.6	C11—C10—C9	119.4 (7)
C1—C2—H2A	108.6	C11—C10—H10A	120.3
N1—C2—H2B	108.6	C9—C10—H10A	120.3
C1—C2—H2B	108.6	C10—C11—C12	118.6 (7)
H2A—C2—H2B	107.6	C10—C11—H11A	120.7
N1—C3—C4	121.0 (9)	C12—C11—H11A	120.7
N1—C3—H3A	119.5	N2—C12—C11	122.9 (6)
C4—C3—H3A	119.5	N2—C12—H12A	118.6
C5—C4—C3	120.1 (10)	C11—C12—H12A	118.6
			
N2—Zn1—O1—C1	55.9 (5)	C2—N1—C3—C4	178.2 (8)
Cl1—Zn1—O1—C1	169.1 (4)	N1—C3—C4—C5	2.5 (14)
Cl2—Zn1—O1—C1	−66.3 (5)	C3—C4—C5—C6	−2.0 (14)
O1—Zn1—N2—C12	−118.6 (5)	C4—C5—C6—C7	−0.2 (13)
Cl1—Zn1—N2—C12	129.2 (5)	C3—N1—C7—C6	−1.6 (11)
Cl2—Zn1—N2—C12	7.2 (6)	C2—N1—C7—C6	179.6 (7)
O1—Zn1—N2—C8	62.7 (6)	C5—C6—C7—N1	2.0 (12)
Cl1—Zn1—N2—C8	−49.4 (6)	C12—N2—C8—C9	−0.7 (11)
Cl2—Zn1—N2—C8	−171.4 (5)	Zn1—N2—C8—C9	178.0 (7)
Zn1—O1—C1—O2	5.2 (9)	N2—C8—C9—C10	−0.6 (14)
Zn1—O1—C1—C2	−173.4 (4)	C8—C9—C10—C11	1.7 (13)
C3—N1—C2—C1	−90.2 (8)	C9—C10—C11—C12	−1.6 (12)
C7—N1—C2—C1	88.6 (8)	C8—N2—C12—C11	0.8 (10)
O2—C1—C2—N1	176.9 (6)	Zn1—N2—C12—C11	−177.8 (5)
O1—C1—C2—N1	−4.3 (9)	C10—C11—C12—N2	0.3 (11)
C7—N1—C3—C4	−0.6 (12)		
